# The versatile defender: exploring the multifaceted role of p62 in intracellular bacterial infection

**DOI:** 10.3389/fcimb.2023.1180708

**Published:** 2023-05-05

**Authors:** Yuhao Zhou, Shucheng Hua, Lei Song

**Affiliations:** ^1^ Department of Respiratory Medicine, Center for Pathogen Biology and Infectious Diseases, Key Laboratory of Organ Regeneration and Transplantation of the Ministry of Education, The First Hospital of Jilin University, Changchun, China; ^2^ State Key Laboratory for Zoonotic Diseases, The First Hospital of Jilin University, Changchun, China

**Keywords:** intracellular bacterial infection, p62 (sequestosome 1(SQSTM1), xenophagy, bacterial protein, microdomain

## Abstract

As a highly conserved, multifunctional protein with multiple domains, p62/SQSTM1 plays a crucial role in several essential cellular activities, particularly selective autophagy. Recent research has shown that p62 is crucial in eradicating intracellular bacteria by xenophagy, a selective autophagic process that identifies and eliminates such microorganisms. This review highlights the various roles of p62 in intracellular bacterial infections, including both direct and indirect, antibacterial and infection-promoting aspects, and xenophagy-dependent and independent functions, as documented in published literature. Additionally, the potential applications of synthetic drugs targeting the p62-mediated xenophagy mechanism and unresolved questions about p62’s roles in bacterial infections are also discussed.

## 1 Introduction

Despite the progress achieved with the development and use of antibiotics and vaccines, the mortality rates associated with infectious diseases remains high. Moreover, antibiotic resistance amongst pathogenic microorganisms has emerged as an alarming global challenge (Rex et al., 2017; Abbara et al., 2022). Intracellular bacterial infections have long been a severe threat to public health. Unlike extracellular microbes that often result in acute infections, intracellular bacteria have the tendency to cause chronic or persistent infections. This type of bacterial infection can remain latent in the host for life, presenting an ongoing threat to the health of the individual (Grant and Hung, 2013). Consequently, research into the mechanisms of intracellular bacterial infection and the host’s defense against such invaders has become a crucial and trending topic in basic biomedical research.

The p62/SQSTM1 protein, a multifunctional protein with a molecular weight of 62-kDa, is found in the cytoplasm in scattered dots or aggregates and can be transported between the cytoplasm and nucleus (Zhang and Costa, 2021). Initially discovered in 1995 for its ability to specifically bind with the src homology 2 domain of p56lck, regulated by phosphorylation of Ser-59 in a phosphotyrosine-independent way, p62 has since been implicated in the pathogenesis of cardiometabolic disease, neurodegenerative disease, malignant tumors, and infectious diseases (Park et al., 1995; Seto et al., 2013; Jeong et al., 2019; Ma et al., 2019; Tang et al., 2021). Additionally, studies have shown that p62 plays a crucial role in xenophagy, a type of selective autophagy that targets intracellular pathogenic microbes and enables their elimination (Knodler and Celli, 2011). This article delves into the role of p62 protein, specifically its involvement in p62-mediated xenophagy, in the infection of various intracellular bacteria like *Salmonella enterica* serovar Typhimurium and *Legionella pneumophila*. In this review, we highlight the current understanding of the mechanisms underlying intracellular bacterial infections and how our innate immune system functions in response to such pathogens (**Table 1**).

**Table 1:** Roles of p62 in intracellular bacterial infection.

Bacterial strain	Mechanisms in bacterial infection	Functions in bacterial survival	Reference
*Mycobacterium tuberculosis*	Mediates xenophagy	Inhibits bacterial survival	(Chai et al., 2019; Berton et al., 2022)
*Mycobacterium tuberculosis*	Facilitating generation of neo-antimicrobial peptides from cytosolic proteins	Inhibits bacterial survival	(Lobato-Marquez et al., 2019)
*Salmonella*	Mediates xenophagy	Inhibits bacterial survival	(Cemma et al., 2011; Mesquita et al., 2012; Heath et al., 2016; Otten et al., 2021)
*Salmonella*	Activates Nrf2-Keap1 pathway	Inhibits bacterial survival	(Ishimura et al., 2014)
*Acinetobacter baumannii*	Mediates xenophagy	Inhibits bacterial survival	(Wang et al., 2016)
*Legionella pneumophila*	Mediates xenophagy	Inhibits bacterial survival	(Omotade and Roy, 2020)
*streptococcus pneumoniae*	Mediates xenophagy	Inhibits bacterial survival	(Shizukuishi et al., 2020)
*Rickettsia parkeri*	Mediates xenophagy	Inhibits bacterial survival	(Borgo et al., 2022)
*Coxiella burnetiid*	Activates Nrf2-Keap1 pathway	No significant impact on bacterial survival observed	(Winchell et al., 2018)
*Shigella flexneri*	Mediates xenophagy targeting Shigella entrapped in septin cages	Inhibits proliferation of bacteria entrapped in septin cages	(Mostowy et al., 2011)
*Shigella flexneri*	Promotes the metabolic activity of intracellular Shigella not entrapped in septin cages	Promots proliferation of bacteria not entrapped in septin cages	(Lobato-Marquez et al., 2019)
*Listeria*	Mediates xenophagy	Inhibits bacterial survival	(Mostowy et al., 2011)
*Burkholderia cenocepacia*	Inhibits BECN1’ function and other autophagy adapter-mediated xenophagy in △F508 macrophages	Promotes bacterial survival	(Abdulrahman et al., 2013)
*Chlamydia trachomatis*	Mediates xenophagy	Inhibits microbial survival	(Wang et al., 2021)

## 2 Microstructure and general role of p62 in selective autophagy

**Figure 1:**
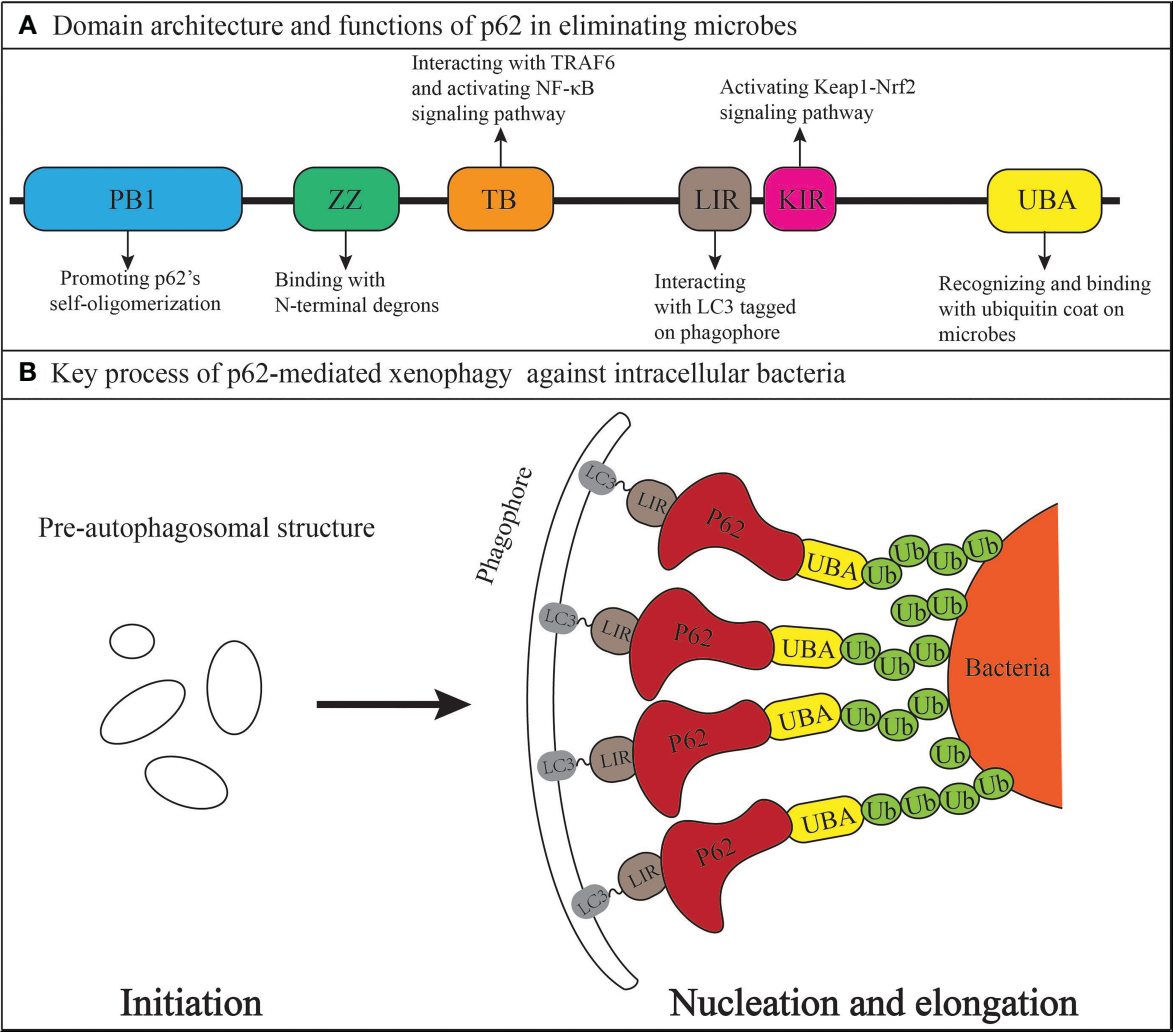
The xenophagy adaptor p62/SQSTM1 **(A)** Domain architecture and anti-bacterial functions. **(B)** Mechanisms in linking the bacteria to a growing phagophore (domains other than the LIR and UBA in the p62 protein are contained within the red region where the word p62 is located).

Comprised of 440 amino acids, p62 is composed of six domains from the N-terminal to C-terminal, including the Phox and Bem1 (PB1) domain (104 amino acids), ZZ-type zinc finger (ZZ) domain (36 amino acids), TRAF6-binding (TB) domain (27 amino acids), LC3-interacting region (LIR) (22 amino acids), Keap1-interacting region (KIR) (6 amino acids), and ubiquitin-associated (UBA) domain (49 amino acids) (Pankiv et al., 2007; Gal et al., 2009; Jiang et al., 2009; Jain et al., 2010; Zhang and Costa, 2021). Notably, p62 has been shown to play a significantly increasing role in multiple intracellular bacterial infections through its involvement in xenophagy against bacteria. The mechanisms behind microbial pathogenesis involve the active invasion of pathogens into host cells or their passive uptake via phagocytosis. Once inside, pathogen-associated molecular patterns such as bacterial surface lipopolysaccharide (LPS) or viral nucleic acid products can alter signaling pathways like nuclear factor kappa B (NF-κB) and mitogen-activated protein kinase, eventually leading to the formation of pre-autophagosomal structures (PAS) facilitated by the endoplasmic reticulum and the Golgi apparatus (Jounai et al., 2007; Xu et al., 2007). Host cells then mark the microbes for destruction by ubiquitination, which generates specific “eat me” signals and enables selective autophagy (Boyle and Randow, 2013). Subsequently, Ser 403 of the UBA domain (aa 391-436) in p62 undergoes phosphorylation by Tank Binding Kinase 1(TBK1), enabling the recruitment of p62 to ubiquitin-coated microbes and initiating its self-oligomerization (Matsumoto et al., 2011; Pilli et al., 2012). During selective autophagy, self-oligomerization of p62 plays a crucial role in delivering ubiquitinated cargos to the autophagy pathway, a process driven by the PB1 domain (Nakamura et al., 2010). The UBA domain in p62 then recognizes and binds to the ubiquitin coat of microbes, while the LIR of p62 interacts with LC3 tagged on the phagophore (PG) originating from PAS. The process is facilitated by the direct binding of N-terminal degrons to the ZZ domain of p62 (Cha-Molstad et al., 2017). Therefore, the microbe is specifically targeted and gradually enveloped by PG via bridging of p62, which leads to the formation of autophagosome. Ultimately, the autophagosome is fused with the lysosome to form the autolysosome in which the microbes are degraded and destroyed by acid hydrolase (Shen et al., 2021; Tripathi-Giesgen et al., 2021). In addition, the TB domain of p62 interacts with TRAF6, leading to the activation of NF-κB signaling pathway which induces further autophagy activation (Min et al., 2018). The domain architecture of p62, functions of each domain, and simplified mechanisms of p62-mediated xenophagy targeting bacteria are summarized in **Figure 1**.

The posttranslational modification of p62 is critical in the process of xenophagy targeting invaders. A study demonstrated that during *Mycobacterium tuberculosis* (Mtb) infection, human protein phosphatase Mg^2+^/Mn^2+^-dependent 1A (PPM1A) inhibit xenophagy via directly dephosphorylating p62 on S403, resulting in impairment of antibacterial effect of host cells. And treatment with a selective PPM1A inhibitor can inhibit the intracellular survival of Mtb in macrophages and in the lungs of infected mice (Berton et al., 2022).

In addition to phosphorylation, ubiquitination of p62 has recently been confirmed to participate in xenophagy against intracellular bacteria. RNF166, a family member of E3 ligases, was found indispensable for recruitment of p62 to *Salmonella* upon infection via directly ubiquitinating p62 at K91 and K189 in a K29- and K33-linked manner, which inhibits bacterial replication through xenophagy but without altering host cell’s autophagic flux (Heath et al., 2016). Notably, studies have revealed that during *Salmonella* infection, the E3 ligase RNF213 may contribute to the formation of a bacterial ubiquitin coat by directly ubiquitinating bacterial LPS. Moreover, RNF213 has been shown to facilitate the recruitment of ubiquitin-dependent autophagy adaptors such as p62 to *Salmonella* and promote subsequent antibacterial xenophagy. These findings suggest that non-proteinaceous substances may also undergo ubiquitylation and recognition in p62-mediated antibacterial xenophagy (Otten et al., 2021).

## 3 The role of p62 in xenophagy against intracellular bacteria

### 3.1 The mechanisms of bacterial proteins involved in p62-mediated xenophagy

There are some typical bacterial proteins involved in host cell’s p62-mediated xenophagy (**Table 2**). As cell surface protein of Mtb, Rv1468c was found to recruit ubiquitin and bind with polyubiquitin via its UBA domain upon Mtb’s infection in macrophages, which promotes p62-mediated xenophagy targeting Mtb (Chai et al., 2019). Meanwhile, researchers also found that deletion of Rv1468c in Mtb reduces colocalizations of Mtb with LC3 and autophagy targeting to Mtb and promotes bacterial intracellular survival.

**Table 2:** Typical bacterial proteins involved in host cell’s p62-mediated xenophagy.

Bacterial protein	Bacterial strain	Mechanisms	Promoting or inhibiting p62-mediated xenophagy	Reference
Rv1468c	*Mycobacterium tuberculosis*	Mediating ubiquitin binding to mycobacterial cell surface	Promoting	(Chai et al., 2019)
Isochorismatase	*Acinetobacter baumannii*	Facilitating recruitment of p62 to bacteria	Promoting	(Wang et al., 2016)
SidE	*Legionella pneumophila*	Phosphoribosyl-ubiquitinating proteins on LCV	Inhibiting	(Omotade and Roy, 2020)
CbpC	*Streptococcus pneumoniae*	Binding with p62 and leading to autophagic degradation of Atg14	Inhibiting	(Shizukuishi et al., 2020)
SseL	*Salmonella typhimurium*	Deubiquitinating substrates targeted by p62	Inhibiting	(Mesquita et al., 2012)
Pat1	*Rickettsia parkeri*	Inhibiting host ubiquitylation machinery	Inhibiting	(Borgo et al., 2022)

It is reported that *Acinetobacter baumannii* could utilize its virulent protein to trigger incomplete autophagy by interfering the fusion of autophagosomes with lysosomes to enhance its survival in infected host cells (An et al., 2019). Meanwhile, Isochorismatase encoded by *Acinetobacter baumannii* was testified to mediate the xenophagy-induced clearance of *Acinetobacter baumannii* via facilitating recruitment of autophagy adaptor p62 and NDP52 to bacteria both *in vitro* and *in vivo* (Wang et al., 2016). However, the specific molecular target of Isochorismatase in the physiological process is unclear.

Although p62-mediated xenophagy plays an essential role in host’s defense against invasive microbes, bacteria have evolved mechanisms to avoid xenophagy through secreting a large cohort of virulence factors called effectors. SidE, an effector protein belonging to the SidE family, help *L. pneumophila* evade xenophagy by disrupting recruitment of p62 to *L. pneumophila*-containing vacuole (LCV) (Omotade and Roy, 2020). Moreover, the study indicates that SidE’s interference with recruitment of p62 to LCV is due to SidE’s phosphoribosyl-ubiquitination of proteins on LCV, and this non-canonical ubiquitination disenables p62 to efficiently recognize bacteria and initiate subsequent xenophagy.

ATG14 is an essential protein in promoting autophagosome-endolysosome fusion in autophagy process (Diao et al., 2015). Sayaka Shizukuishi et al. reported that during *streptococcus pneumoniae* infection, CbpC, which belongs to pneumococcal cell surface proteins, could bind to p62 and act as a decoy for autophagic degradation of Atg14, thus suppressing host’s xenophagy targeting intracellular pneumococci and promoting bacterial survival within host cells (Shizukuishi et al., 2020).

SseL, a virulent protein originating from *Salmonella*’s type 3 secretion systems(T3SS), was demonstrated to inhibit host cell’s xenophagy and promote *Salmonella*’s replication (Mesquita et al., 2012). Mechanistically, SseL deubiquitinates autophagic substrates targeted by p62 and induced by *Salmonella*, which inhibits the recruitment of p62 and LC3 to SCV-associated aggregates.

Belonging to obligate intracellular bacteria, *Rickettsia parkeri* could evade host cell’s xenophagy and easily spread from cell to cell (Helminiak et al., 2022). Encoded by all sequenced Rickettsia species, patatin-like phospholipase A2 enzyme (Pat1) was found to enable *Rickettsia parkeri*’s evasion of recognition by p62-mediated xenophagy through avoidance of polyubiquitin recruitment (Borgo et al., 2022).

### 3.2 The interaction between p62 and Nrf2-Keap1 pathway in bacterial infection

The antimicrobial function of p62 is not limited to xenophagy, but may also involve the Nrf2 (nuclear factor erythroid 2-related factor 2)-Keap1 (kelch-like ECH-associated protein 1) pathway. Previous studies have demonstrated that upon sequestration of p62 to *Salmonella* and its subsequent oligomerization, Ser351 of the KIR domain (amino acids 346-359) is phosphorylated, leading to recruitment of Keap1 onto p62-positive microbes. This results in the activation of Nrf2, which translocate into the nucleus and induces the transcription of cytoprotective genes, including scavenger receptors and enzymes involved in the pentose phosphate pathway to generate nicotinamide adenine dinucleotide phosphate. The scavenger receptors increase the number of bacteria in phagosomes, whereas the enzymes elevate the level of reactive oxygen species in phagosomes through activation of NADPH oxidase, both of which aid in eliminating intracellular bacteria (Harvey et al., 2011; Bonilla et al., 2013; Ishimura et al., 2014).

In turn, certain bacteria can exploit host Nrf2-Keap1 pathway via modifying p62. A study showed that during *Coxiella burnetiid* infection, instead of binding with pathogen-containing vacuole directly, p62 is recruited to the vicinity of the parasitophorous vacuole in which *Coxiella burnetiid* resides, which is independent of UBA domain or LIR of p62. In addition, the levels of phosphorylated p62(S349) were remarkably increased throughout infection, coincident with activation of Nrf2-Keap1 pathway (Ichimura et al., 2013; Winchell et al., 2018). However, downregulating p62 seems to have no significant impact on bacterial growth in THP-1 cells, which indicates that p62 is not absolutely required for certain bacterial intracellular replication and is utilized by bacteria during their intracellular growth (Winchell et al., 2018).

### 3.3 The interaction between p62 and NDP52 through xenophagy in different bacterial infection

p62-mediated xenophagy in intracellular bacterial infection may be affected by another autophagy adapter NDP52. Septins, a kind of conserved GTP-binding proteins, form cages around intracytosolic *Shigella* but not *Listeria* to restrict bacterial proliferation (Mostowy et al., 2010). During *Shigella*’s infection, p62 and NDP52 are recruited interdependently to the *Shigella*-containing septin cages and regulate each other’s xenophagic activity (Mostowy et al., 2011).

However, when host cells are under *Listeria*’s invasion, p62 and NDP52 can be recruited independently of each other. Likewise, another research suggested that though sharing similar kinetics of recruitment to *Salmonella*, p62 and NDP52 are recruited independently of one another to target different microdomains surrounding bacteria to facilitate xenophagy (Cemma et al., 2011).

### 3.4 The rare role of p62 in promoting intracellular bacterial proliferation

In fact, p62 does not always play an antimicrobial role in all cases of intracellular bacterial infection. Usually accompanied by *Burkholderia cenocepacia* infection, cystic fibrosis (CF) is caused by mutations in the cftr gene encoding the cystic fibrosis transmembrane conductance regulator (CFTR), which usually results in a deletion of phenylalanine at position 508 (△F508) (Luciani et al., 2011). The △F508 cell’s normal processing of the CFTR protein to the epithelial cell surface is hindered, resulting in an aggresome-prone protein that forms intracellular aggregates which sequester autophagy molecule beclin1 (BECN1) and further inhibit the early stages of autophagosome formation (Deretic, 2010; Luciani et al., 2010; Luciani et al., 2011). Surprisingly, the researchers found that knockdown of p62 in △F508 macrophages increased *B. cepacia*’s colocalization with LC3 and inhibited the growth of bacteria, and vice versa (Abdulrahman et al., 2013). Mechanistically, downregulation of p62 liberated BECN1 from aggregates, allowing its redistribution in the cytosol and recruitment by the *B. cepacia*-containing vacuole and recuperating autophagy in △F508 macrophages. Furthermore, depletion of p62 enables autophagy adapter NDP52 and NBR1 to facilitate the delivery of *B. cepacia* to autophagosomes and inhibit the bacteria’s growth.

Moreover, while p62 has been found to target intracellular *S. flexneri* that are trapped in septin cages to initiate xenophagy and curb bacterial replication, recent studies also suggest that p62 can potentially enhance the proliferation of free *S. flexneri* that are not entrapped in septin cages (Mostowy et al., 2011; Lobato-Marquez et al., 2019). Additionally, p62 has been shown to boost the metabolic activity of intracellular *Shigella* not trapped in septin cages. However, the exact mechanism by which p62 promotes the metabolism of free *Shigella* requires more systematic exploration.

### 3.5 Indirect function of p62 in facilitating xenophagy

p62 may facilitate clearance of invaders through producing substances with antibacterial activity. For example, upon Mtb invaded host cells, p62 could capture certain cytosolic proteins, such as ribosomal protein rpS30 precursor FAU and ubiquitin, and then deliver them from the cytosol into conventional autophagic organelles, leading to their degradation by proteolysis into smaller peptides which possess antimicrobial properties (Ponpuak and Deretic, 2011). Subsequently, these vesicles containing small antimicrobial peptides are transported into autophagosomes in which Mtb resides and kill them, which indirectly contributes to xenophagy.

## 4 Potential drugs against intracellular bacterial infection based on p62

To explore a new effective pharmaceutical means to eliminate various drug-resistant intracellular bacteria, Yoon Jee Lee et al. developed and synthesized chemical p62 agonists targeting the N-degron pathway and facilitating p62-mediated xenophagy under microbes’ invasion (Lee et al., 2022). They found that these p62 agonists rescue Hela cell’s autophagic activities from suppression by S. Typhimurium. Mechanistically, to promote xenophagy targeting S. Typhimurium, the p62 agonists bind directly to the ZZ domain of p62, facilitating transportation of the pathogens to the autophagosome. Furthermore, the p62 agonists was confirmed to suppress the growth of Mtb, Gram-negative *Escherichia coli* and the Gram-positive *Streptococcus pyogenes* through xenophagy, suggesting its potential as a drug in killing a broad range of pathogenic bacteria. More encouragingly, in addition to its antimicrobial effect, the anti-inflammatory effect of p62 agonists were also observed both *in vitro* and *in vivo*. Interestingly, the antimicrobial efficacy is independent of rapamycin-modulated core autophagic pathways and is synergistic with the reduced production of inflammatory cytokines. Collectively, manual chemical activation of universally conserved p62-dependent xenophagy could be a promising treatment for infectious diseases caused by various bacterial strains. Previously, cytokine TNF-α was confirmed to stimulate the activity of p62-mediated xenophagy targeting intracytosolic *Shigella* and *Listeria* (Mostowy et al., 2011). However, whether TNF-α will affect overall autophagy flux which is required during normal metabolic activity of cell needs further investigation.

Apart from its role in eliminating bacteria through xenophagy, p62 is also pivotal in reducing inflammasome activity and mitigating acute pulmonary inflammation resulting from *L. pneumophila*. This is achieved through the direct binding of p62 to NLR proteins of the inflammasome, further underscoring the potential of p62 as a therapeutic target in managing the severity of Legionnaires’ disease (Ohtsuka et al., 2014).

## 5 Mysteries about roles of p62 in intracellular bacterial infection

Although much progress has been made regarding roles of p62 in host’s antibacterial mechanisms, some mysteries therein still need to be unveiled. For example, it is demonstrated that *Chlamydia trachomatis* infection of HeLa cells could induce p62-dependent xenophagy 24h post-infection. However, a decrease of xenophagy levels at 48h post-infection was observed in study, indicating a defect in sustained autophagy initiation with limited p62 protein after lysosomal degradation of autophagosomes (Wang et al., 2021). Moreover, downregulation of p62 did not influence the morphology and number of *C. trachomatis* infecting HeLa cells, suggesting that there must be other antibacterial mechanisms other than xenophagy which play significant roles in host cell clearance of *C. trachomatis*.

As a cytoplasmic protein, the role of p62 outside the cells is rarely investigated. A study focusing on sepsis indicated that treatment of macrophages and monocytes with lipopolysaccharide phosphorylated intracellular p62 at Ser403 and induced pyroptosis which enable passive release of p62. Furthermore, extracellular p62 binds directly with insulin receptor (INSR) on cell surface to activate downstream NF-κB-dependent metabolic programming, which mediates bacterial septic death in mice (Zhou et al., 2020). However, whether extracellular p62 could facilitate or inhibit intracellular bacterial growth still needs to be further considered and explored.

## 6 Summary

To sum up, functioning as a cytoprotective protein in most cases, p62 defends against pathogen’s invasion mainly in a xenophagy-dependent way during intracellular bacterial infection. However, some mysteries regarding roles of p62 in bacterial infection still wait to be explored, and uncovering these mysteries will help us further understand the mechanisms of bacterial infection and develop targeted and effective antibacterial drugs.

## Author contributions

YZ, SH, and LS conceived and pursued the design of the review. YZ and LS wrote the manuscript. YZ, SH, and LS critically revised the manuscript. All authors read the manuscript. All authors substantially contributed to the discussions of content and reviewed or edited the manuscript before final submission.
